# Improving the Digestibility of Plant Defensins to Meet Regulatory Requirements for Transgene Products in Crop Protection

**DOI:** 10.3389/fpls.2020.01227

**Published:** 2020-08-14

**Authors:** Kathy Parisi, Simon Poon, Rosemary F. Renda, Gurinder Sahota, James English, Nasser Yalpani, Mark R. Bleackley, Marilyn A. Anderson, Nicole L. van der Weerden

**Affiliations:** ^1^Department of Biochemistry and Genetics, La Trobe Institute for Molecular Science, Bundoora, VIC, Australia; ^2^Department of Animal, Plant and Soil Sciences, School of Life Sciences, La Trobe University, Bundoora, VIC, Australia; ^3^Maxygen LLC, Sunnyvale, CA, United States; ^4^Corteva Agriscience, Agriculture Division of DowDuPont, Johnston, IA, United States; ^5^Department of Biology, University of British Columbia, Kelowna, BC, Canada

**Keywords:** antimicrobial protein, antifungal, plant defensin, simulated gastric fluid, simulated intestinal fluid, regulations, transgenic plants, allergenicity

## Abstract

Despite the use of chemical fungicides, fungal diseases have a major impact on the yield and quality of plant produce globally and hence there is a need for new approaches for disease control. Several groups have examined the potential use of antifungal plant defensins for plant protection and have produced transgenic plants expressing plant defensins with enhanced resistance to fungal disease. However, before they can be developed commercially, transgenic plants must pass a series of strict regulations to ensure that they are safe for human and animal consumption as well as the environment. One of the requirements is rapid digestion of the transgene protein in the gastrointestinal tract to minimize the risk of any potential allergic response. Here, we examine the digestibility of two plant defensins, NaD1 from *Nicotiana alata* and SBI6 from soybean, which have potent antifungal activity against major cereal pathogens. The native defensins were not digestible in simulated gastrointestinal fluid assays. Several modifications to the sequences enhanced the digestibility of the two small proteins without severely impacting their antifungal activity. However, these modified proteins did not accumulate as well as the native proteins when transiently expressed *in planta*, suggesting that the protease-resistant structure of plant defensins facilitates their stability *in planta*.

## Introduction

Fungal infections have a major impact on the yield and quality of produce from plants and hence their control is essential for global food security (Pennisi, 2010). Fungal diseases destroy up to 20% of the world’s crops and contamination by fungal toxins causes serious health issues in humans and animals (Normile, 2010; Bebber and Gurr, 2015). Furthermore, more virulent strains of fungi are developing and spreading globally, leading to severe disease outbreaks and threatening famine in developing countries (Thornton and Wills, 2015). Several practices have been developed for control of fungal diseases in agriculture. They include use of chemical fungicides (Damicone and Smith, 2009), rotation of crops (Peng et al., 2014) and use of germplasm with different resistance genes (R-genes) (Sokolova et al., 2011; Van De Wouw et al., 2014). Despite the introduction of these measures, there are still substantial crop losses from fungal diseases worldwide.

Plants have a number of unique defense mechanisms including physical barriers to pathogen invasion as well as a wide range of secondary metabolites and antimicrobial proteins (AMPs) for protection against pests and diseases (Tavares et al., 2008). Plant defensins are one of the largest families of AMPs and are generally very effective against a broad spectrum of fungal pathogens of both plants and humans (Van Der Weerden and Anderson, 2013; Parisi et al., 2019; Sathoff et al., 2019). All plant defensins contain one α-helix and three antiparallel β-strands stabilized by four disulfide bonds to make up the tertiary structure termed the cystine stabilized αβ (Csαβ) motif (Broekaert et al., 1995; Bulet et al., 1999). This architecture is highly stable to proteases, and extremes of pH and temperature (Woytowich and Khachatourians, 2001). Plant defensins are further divided into class II defensins, which are produced from precursors that have a C-terminal prodomain (CTPP) that directs them to the vacuole where the CTPP is removed, and class I defensins that do not have a CTPP and are directed to the apoplast (Parisi et al., 2019).

Several groups have produced transgenic plants expressing defensins that have enhanced resistance to fungal diseases either in the greenhouse or in the field (Iqbal et al., 2019). They include transgenic cotton plants expressing NaD1, a defensin from the ornamental tobacco, that displayed enhanced resistance to fungal wilts over 3 years of field trials (Gaspar et al., 2014), transgenic potatoes expressing the alfAFP defensin from alfalfa (*Medicago sativa*) which were resistant to the fungal pathogen *Verticillium dahliae* (Gao et al., 2000) and transgenic rice constitutively expressing the wasabi defensin which was resistant to rice blast (Kanzaki et al., 2002). Despite these promising results, none have progressed into commercial development. One of the reasons for this is the difficulty in obtaining regulatory approval for new transgenes.

An international regulatory framework has been established that requires compliance with regulatory procedures for research, product development, and distribution of genetically modified plant products (Nap et al., 2003; Jaffe, 2004; Falck-Zepeda et al., 2012). Regulators stringently monitor any potential risks a GM crop may pose to human health and the environment. One of these risks is the potential for a GM crop to induce allergic sensitization and/or subsequent allergic reactions (Misra et al, 2012) *via* the protein encoded by the transgene or from changes in the levels of existing protein allergens when a transgene is present (Kimber and Dearman, 2002). To minimize this risk, regulators ensure that proteins encoded by transgenes are not allergens.

Thus, the regulations stipulate that the products of transgenes must be non-immunogenic and rapidly degraded in the digestive system to fragments less than 3.5 kDa. To meet these regulations, a weight-of-evidence approach is used to assess the risk of allergenicity of an introduced protein, because no single assay or property can distinguish an allergen from a non-allergen (FAO/WHO, 2001). This approach encompasses the source of the transferred gene, size of the encoded protein, any sequence identity of that protein with known allergens, the prevalence of the protein in foods and the stability of the protein both during food processing and in gastric juices (digestive stability). The stability of a protein in the presence of digestive proteases of the gastrointestinal (GI) tract is assessed using the Simulated Gastric Fluid (SGF) assay and/or the Simulated Intestinal Fluid (SIF) assay (Fu et al., 2002; Herman et al., 2006).

Here we demonstrate that two plant defensins, NaD1 from the ornamental tobacco *Nicotiana alata* and SBI6 from soybeans, are stable in simulated gut and intestinal fluid assays and hence would not pass the digestion criteria for a transgene product. We describe modifications to these plant defensins that markedly increase digestibility without severely impacting their antifungal activity. However, these modified defensins failed to accumulate to the same levels as unmodified defensins when transiently expressed *in planta*.

## Materials and Methods

### Predicting Potential Allergenicity

The primary sequence of NaD1 was analyzed for allergen sequence similarity using the allergen databases; Structural Database of Allergenic Proteins (Ivanciuc et al., 2003), AlgPred database (Saha and Raghava, 2006), and AllergenOnline (Goodman et al., 2016). The sequence of NaD1 was searched for six to eight contiguous amino acid matches with known allergic proteins using all three databases. Mapping of IgE specific epitopes was performed using the AlgPred database.

#### Extraction and Purification of NaD1

The NaD1 defensin was extracted and purified from *N. alata* flowers as described by van der Weerden and colleagues (Van Der Weerden et al., 2008). The protein was purified further using reversed phase high-performance liquid chromatography (RP-HPLC) with a C8 Agilent column as described previously (Lay et al., 2003). The protein concentration was determined using the BCA protein assay (Pierce).

#### Strains and Vectors

The SHuffle^®^ T7 *E. coli* strain (New England BioLabs, United States) was used to express the recombinant variant defensins. The SHuffle^®^ T7 *E. coli* cells (F´ *lac*, *pro*, *lacIQ*/Δ(ara-leu)7697 araD139 fhuA2 lacZ::T7 gene1 Δ(phoA)PvuII phoR ahpC* galE (or U) galK λatt::pNEB3-r1-*cDsbC* (Spec^R^, *lacIq*) *ΔtrxB rpsL150*(Str^R^) *Δgor Δ(malF)3*, New England BioLabs, United States) are designed for production of proteins containing disulfide bonds in the cytoplasm. A single amino acid variant library of the NaD1-NaD2 chimeric defensin, NaD1-L1B-HRFKGP, was synthesized and cloned into the *p*MAXY3142 expression plasmid (Bleackley et al., 2016). The parent NaD1-NaD2 chimeric defensin has the entire NaD1 sequence apart from the loop 1B sequence which has been replaced with the loop 1B sequence (HRFKGP) from NaD2 (Bleackley et al., 2016). The *p*MAXY3142 plasmid expressed the proteins of interest as a fusion with the maltose binding protein for increased stability and incorporated an N-terminal hexa-histidine (6xHis) tag for purification by immobilized metal ion affinity chromatography (IMAC). Expression was controlled by the lac operon and induced by isopropyl β-D-1-thiogalactopyranoside (IPTG) (Di Guana et al., 1988) in the presence of 15 μg/ml kanamycin, 34 μg/ml chloramphenicol, 100 μg/ml ampicillin, and 10 μg/ml tetracycline (KCAT). The maltose binding protein was cleaved from the fusion with Factor Xa (Maina et al., 1988; Jenny et al., 2003).

The pHUE vector was used with chemically competent SHuffle^®^ T7 *E. coli* cells to express the defensin variants. The pHUE vector is a histidine-tagged, ubiquitin fusion expression vector that allows for purification of the fused protein by IMAC (Baker et al., 2005). It has an inducible T7 RNA polymerase promoter, expresses the lac repressor lacI^q^ and confers resistance to ampicillin. The recombinant defensins were cleaved from the ubiquitin using the deubiquitylating enzyme Usp2cc (Baker et al., 2005). The yield of recombinant protein was 0.36–2.5 mg/L using the SHuffle^®^ T7 *E. coli* strain.

#### Screening the NaD1-L1B-HRFKGP Variant Library

##### Design of a Library of NaD1-L1B-HRFKGP Variants With Single Amino Acid Substitutions

An alignment of 165 defensin sequences from a range of plants was used to identify the most and least highly conserved residues in plant defensins. This was used to inform the design of the NaD1-L1B-HRFKGP single amino acid substitution library. DNA encoding each of the 171 single amino acid variants (1 μl) was transformed into 20 μl of *E. coli* Rosetta-gami ™ B (DE3) cells (Novagen) in individual wells in PCR strips (SSI, Interpath) using a standard heat shock method (Sambrook et al., 1989).

##### Expression and Purification of the NaD1-L1B-HRFKGP Variant Library

Each variant (5 µl) was transferred into a well of a microtitre plate containing 200 μl of 2YT medium with KCAT antibiotics. The plates were incubated at 37°C with agitation (620 rpm) overnight. The Agilent Bravo 96 channel automated liquid handling platform (Agilent Technologies) was then used to transfer 50 μl of culture into the wells of 48-well deep well plates (Axygen Scientific) containing 2.5 ml of 2YT media with KCAT. The plates were covered with breathable membranes (B100, Interpath) and incubated at 37°C in the plate multitron incubator (In vitro Technologies) with agitation (600 rpm) until the optical density at 600 nm (OD_600_) was between 0.6 and 0.8. Recombinant protein expression was induced by the addition of IPTG (1 mM final concentration) and incubation at 16°C overnight with agitation (600 rpm). The cells were harvested by centrifugation at 3220*g* for 10 min at 4°C. Supernatants were discarded, and the pellets resuspended in 275 μl of lysis buffer (10 mM Tris-HCl pH 8.0, 100 mM MgCl_2_, 10 mg/ml lysozyme, and 1 mg/ml endonuclease). The resuspended cells were lysed by freezing at -80°C and thawing in a 37°C incubator (twice) before incubation at 37°C for 1 hour. Cell debris was removed by centrifugation at 3220*g* for 20 min.

The NaD1-L1B-HRFKGP variant proteins were purified from cell lysates using 96-well filter plates preloaded with commercially available nickel nitriloacetic acid resin (Amintra Ni-NTA resin, DKSH) as described by Schafer and colleagues (Schafer et al., 2002) using the Agilent Bravo liquid handling robot. Cleavage of the NaD1-L1B-HRFKGP variant proteins from the maltose binding protein and his-tag was accomplished *via* incubation with two units of Factor Xa (Restriction grade, bovine plasma, Merck Millipore) overnight at room temperature.

#### Modification of NaD1 to Improve Digestibility to Pepsin

##### Site Directed Mutagenesis of NaD1

Mutations designed to improve pepsin cleavage of NaD1 included i) introduction of the pepsin cleavage sequence from lactoferrin, RAFALE, into loop 1B, ii) the K28F substitution which introduced an FF motif into loop 4, iii) substitution of cysteine residues 3 and 47 with serine residues to remove the disulfide bond between the N- and C- termini and iv) combinations of these variations. The pHUE-NaD1 construct was used as the mutagenesis template. The primers used to introduce the site directed mutations were synthesised and purified by GeneWorks Pty Ltd (Australia/New Zealand) with 5’ phosphorylation. Mutations were generated by PCR amplification using Phusion^®^ Hot Start Flex as described in (Bleackley et al., 2016). The PCR amplicons were evaluated using 1.2% agarose gel electrophoresis and visualized with SYBR safe (LifeTechnologies, Australia) prior to purification using the QIAquick^™^ PCR purification kit (Qiagen, Germany) or the Wizard^®^ SV Gel and PCR Clean Up System (Promega, United States) according to their respective manufacturer’s instructions. The amplicons (600 ng with 6.4 pmole of nested primer) were then sequenced by the Australian Genome Research Facility (Melbourne, Australia). DNA, with the confirmed mutations, was transformed into SHuffle^®^ T7 Competent *E. coli* cells for protein expression consistent with the manufacturers’ instructions. The expressed protein was purified using nickel affinity chromatography and the protein was subsequently cleaved from the histidine tagged ubiquitin using Usp2cc enzyme. The protein was further purified using reverse phase high performance liquid chromatography with a Zorbax C8 semi-preparative column (Agilent). The variants were confirmed for correct molecular mass using mass spectrometry (MALDI-TOF, Bruker).

#### Modification of SBI6 to Improve Digestibility With Pepsin

##### SBI6 Extraction and Purification

SBI6 was extracted from soybean seeds as described for NaD1 extraction from flowers (Van Der Weerden et al., 2008).

##### Site Directed Mutagenesis of SBI6

The pHUE-SBI6 construct was used as the template for site directed mutagenesis to introduce the RAFALE sequence into loop 1 and the K28F, C3S, and C47S modifications into the SBI6 backbone. This was performed as described for the NaD1 modifications. Plasmids containing the required sequence were transformed into Shuffle T7 *E. coli* cells, expressed and purified as described for the NaD1 variants.

#### Digestibility of Defensins With Pepsin and Trypsin

##### Pepsin and Trypsin Cleavage Site Prediction

A prediction of the potential pepsin and trypsin cleavage sites in NaD1 and the defensin variants was obtained using the ExPASY (Gasteiger et al., 2005) peptide cutter software (www.expasy.org/peptide_cutter/) with the pepsin pH 1.3 and trypsin parameters respectively.

##### Simulated Gastric Fluid (SGF) Pepsin Digestion Assay

The SGF assay was performed as described by Tong-Jen Fu and colleagues (Fu et al., 2002) with some modification. Tests were performed in 500 µL of SGF (200 mg NaCl, 10 units pepsin/µg test protein, pH 1.2) in glass vials with stir bars that were set on a submersible stir plate (2mag MIX drive, Sigma Aldrich) in a 37°C water bath for continuous stirring of the solutions throughout the assay. After 2 min preincubation, the assay was started by the addition of 25 µl of test protein (5 mg/ml) to each vial containing SGF, SGF minus pepsin or ultrapure water. Bovine serum albumin (minimum 98%, A-7030, Sigma Aldrich) (5 mg/ml in ultrapure water) was used as the positive control for pepsin digestion. Protein samples were analyzed for degradation by SDS PAGE followed by staining with RAPIDStain (Calbiochem) for 1 h after intense washing with ultrapure water.

##### Simulated Intestinal Fluid (SIF) Trypsin Digestion Assay

The SIF assay was performed as described by Tong-Jen Fu and colleagues with modifications. Aliquots (320 µl) of each of SIF (60 mM KH_2_PO_4_, pH 7.5, 12 mg/ml pancreatin), SIF minus pancreatin and ultrapure water were added to individual glass vials with stir bars on a submersible stir plate (2mag MIX drive, Sigma Aldrich) in a 37°C water bath for 2 min before the addition of 40 µl of test protein (5 mg/ml) to each vial. Azoalbumin (A-2382, Sigma Aldrich) at a final concentration of 10 mg/ml in ultrapure water was used as the positive control for protease digestion. Protein degradation was assessed by SDS PAGE as described above.

##### Sequential SGF (Pepsin) and SIF (Trypsin) Digestion Assays

This assay was performed to monitor the digestion of the test protein in SGF followed by SIF. The SGF was prepared as above and 380 µl was pre-incubated at 37°C for 2 min in a glass vial on the submersible stir plate. The reaction was initiated by the addition of 20 µl of test protein (5 mg/ml). A sample (100 µl) was removed at 30 and 60 min and neutralized with 35 µl of 200 mM Na_2_CO_3_, pH 11. The remaining reaction mix was neutralized with 40 µl of 0.5 M NaOH. The test protein was subsequently treated with trypsin in SIF by addition of 160 µl of concentrated SIF (125 mM KH_2_PO_4_, 25 mg/ml of pancreatin). A sample (200 µl) was removed at 0.5 and 30 min and inactivated at >75°C for 10 min. Proteolysis was assessed by SDS PAGE as described above.

#### Mass Spectrometry of Proteolysed Fragments

##### Determination of Fragment Size Post-Incubation in SGF

Digestion products from the SGF assays were analyzed by Matrix-assisted laser desorption/ionization time of flight (MALDI TOF) mass spectrometry. Samples were pre-incubated with 0.5 μl of 40 mM DTT for 20 min on the ground steel plate to reduce disulfide bonds. The samples were subsequently mixed with 2,5-dihydroxybenzoic acid (DHB) and the mass to charge ratio was measured for each sample using a Bruker Daltonics Ultraflex III MALDI-TOF/TOF Mass Spectrometer.

#### Antifungal Activity of Each Variant on Two Major Plant Pathogens

The *C. graminicola isolate* (United States, Carroll-1A-99) and the *F. graminearum* isolate (73B1A) were isolated from *Zea mays* and provided by Pioneer Hi-Bred International, Inc. (Johnston, Iowa, USA). Spores (*F. graminearum* and *C. graminicola*) were isolated from sporulating cultures growing on half-strength potato dextrose broth agar (½ PDA). Antifungal assays were performed as described by van der Weerden and colleagues (Van Der Weerden et al., 2008). The plates were incubated in the dark at 25°C for 24 h for *F. graminearum* and 40 h for *C. graminicola* and growth was measured using a microtitre plate reader (SpectraMax Pro M5e, Molecular Devices) at 595 nm using the nine well scan at 0, 24, and 40 h. The nine well scan reads each well at nine specific positions and an average of the nine reads is provided.

#### Accumulation of Digestible NaD1 Variants *in Planta*

DNA encoding NaD1, NaD1-L1B-HRFKGP, NaD1-C3S-C47S, or NaD1-L1B-RAFALE (± the C-terminal propeptide of NaD1) as well as the p19 suppressor of gene silencing (Scholthof, 2007) was cloned into a vector containing the cauliflower mosaic virus 35S promoter and terminator sequences (Tabe et al., 1995) before transfer into the binary vector pBIN19 (Bevan, 1984). The pBIN19 constructs were then transformed into *Agrobacterium tumefaciens* (strain LBA4404) by electroporation. The defensin genes were transiently co-expressed (1:1) with p19 by agroinfiltration of bush bean cotyledons (*Phaseolus vulgaris* cv. Royal Burgundy) as described previously (Poon et al., 2018). Infiltrated plant tissue was harvested after 5 days. It was then snap-frozen in liquid nitrogen and ground in a mixer mill before proteins were extracted for analysis by NaD1 ELISA (Gaspar et al., 2014). Accumulation of each defensin variant was determined using an ELISA with the NaD1 antibody together with a standard curve of the respective protein. Plant extracts were diluted between 1/2000 and 1/300,000. Agroinfiltration with empty vector was used as a control. The mean transient expression in ppm for each construct was compared using Tukey’s HSD, IBM SPSS Version 25.0. P values for all comparisons are reported in **Supplementary Figure 2**.

## Results

### *In Silico* Analysis of the NaD1 and SBI6 Protein Sequences to Identify Potential Allergenicity

**Figure 1:**
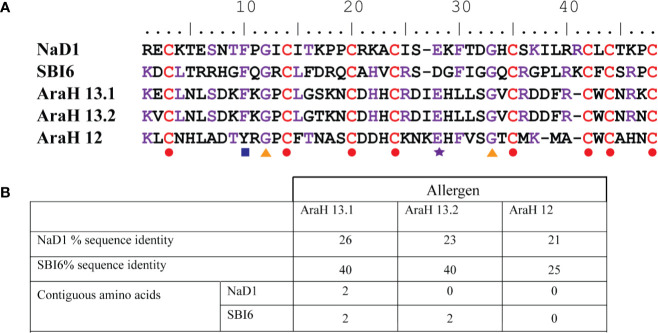
Sequence identity of defensins NaD1 and SBI6 with the three peanut defensins. **(A)** Sequence alignment. The conserved residues are indicated with colored shapes below the alignment. Residues in NaD1 or SBI6 that are identical with either of the peanut defensins are depicted in purple, the cysteines that define the defensin fold are depicted in red. The eight cysteines, glycines 12 and 33, glutamic acid 28, and the aromatic residue at position 10 are strictly conserved or common in all defensins (Parisi et al., 2019) **(B)** List of the percent sequence identity between NaD1 and SBI6 and the peanut defensins. The maximum number of contiguous residues is also listed.

*In silico* analysis of the protein sequences of NaD1 and SBI6 revealed no stretches of eight or more consecutive amino acids with sequence identity to known allergens using three databases (SDAP, AlgPred and Allergenonline). However, sequence identity to peanut defensins, which are potential food allergens, was detected. SBI6 and NaD1 share sequence identities of 40%, 40%, and 25% and approximately 26%, 23%, and 21% with the peanut defensins Arah 13.1, Arah 13.2, and Arah 12 respectively (**Figure 1B**). Most of the sequence identity is from the conserved cysteines and two glycines that define the defensin structure and not from contiguous amino acids (**Figure 1A**). The 80 amino acid sequence alignments, required by FAO/WHO, were not performed because plant defensins are less than 60 residues in length. *In silico* analyses of IgE epitopes using the AlgPred database revealed that NaD1 and SBI6 have no experimentally proven IgE binding epitopes.

### Modifications to NaD1 and SBI6 Improve Digestibility to Pepsin

**Figure 2:**
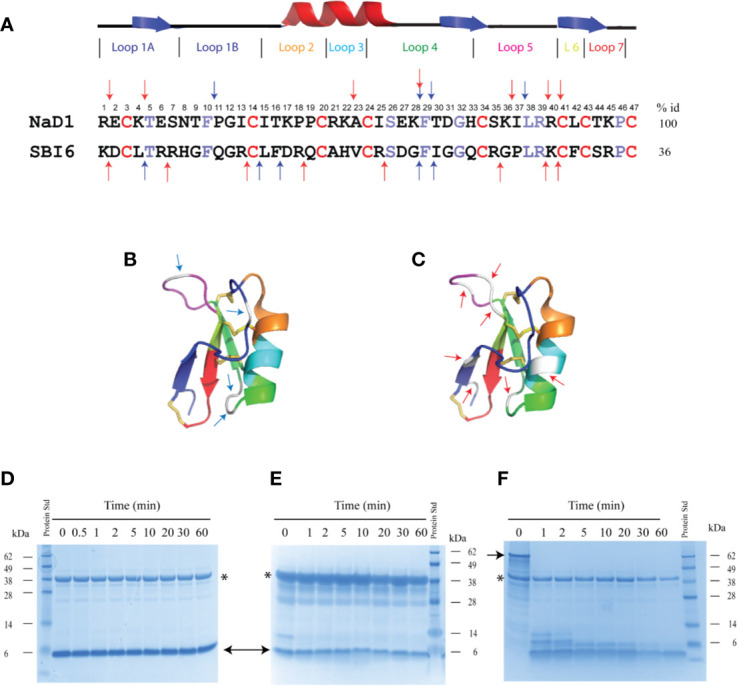
Sequence alignment of SBI6 and NaD1 with predicted pepsin cleavage sites that are not cleaved with pepsin in the simulated gastric fluid assay. **(A)** A sequence alignment of NaD1 and SBI6 with the conserved cysteine residues highlighted in red and the other conserved residues between NaD1 and SBI6 highlighted in purple. Blue arrows indicate the predicted pepsin cleavage sites and red arrows highlight the predicted trypsin cleavage sites. **(B)** Predicted pepsin cleavage sites indicated on the three-dimensional structure of NaD1 (PDB 1MR4) **(C)** Predicted trypsin cleavage sites indicated on the three-dimensional structure of NaD1. **(D–F)** The two defensins and BSA were each incubated in simulated gastric fluid for up to 60 min. Samples were collected at various time point and analysed by SDS-PAGE. No breakdown of NaD1 **(D)** or SBI6 **(E)** was evident. BSA **(F)** was completely digested by pepsin within 1 min. Size markers are indicated on the side of the gels. Pepsin is marked with an asterisk and the two defensins and BSA with arrows.

Two defensins that differ markedly in sequence were chosen for the digestibility studies. They were the class II defensin NaD1 from the ornamental tobacco *Nicotiana alata* and the class I defensin SBI6 from soybean. SBI6 from soybeans shares 36% sequence identity with NaD1 (**Figure 2A**). NaD1 has four predicted pepsin cleavage sites and seven predicted trypsin cleavage sites whereas SBI6 has five and eight cleavage sites respectively (**Figures 2A–C**).

**Figure 3:**
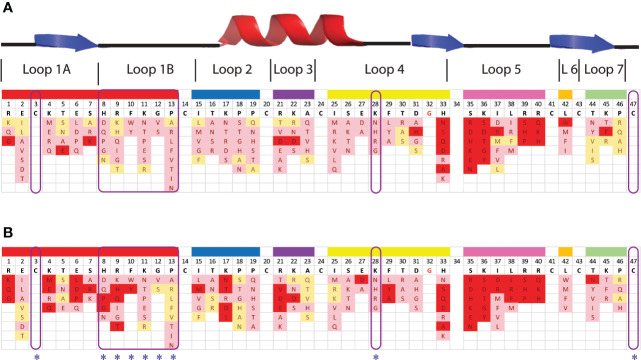
Effect of amino acid variations in the loop regions of NaD1-L1B-HRFKGP on the activity of NaD1-L1B-HRFKGP against *F. graminearum* and *C. graminicola*. Summary of single amino acids variants and their antifungal activity against **(A)** *F. graminearum* and **(B)** *C. graminicola*. Red represents substitutions that abolished antifungal activity at the concentrations tested. Pink represents decreased antifungal activity relative to wild type. Yellow represents no decrease in antifungal activity compared to wild type NaD1-L1B-HRFKGP. The blue asterisks highlight the residues that were modified to enhance pepsin cleavage.

Despite the presence of potential pepsin cleavage sites, both defensins were stable in the SGF assay for more than 60 min (**Figures 2D, E**). Therefore, we introduced pepsin cleavage sites into more accessible loop regions of the NaD1 structure to enhance digestibility. Before we did this, we conducted a single amino acid screen on NaD1-L1B-HRFKGP, a variant of NaD1 with improved antifungal activity, to identify regions of the defensin that could be modified without severely impacting the activity against the plant pathogens *F. graminearum* (FGR) and *C. graminicola* (CGR) (**Figure 3**).

NaD1-L1B-HRFKGP is a chimeric defensin that was produced by substituting the loop 1B region of the plant defensin NaD1 from *N. alata* with the same region from NaD2, another defensin from the same plant (Bleackley et al., 2016). The single amino acid variants of NaD1-L1B-HRFKGP were tested in growth assays with *F. graminearum* and *C. graminicola*. The screen revealed that most of the residues in loop 5 are essential for antifungal activity against both pathogens as most substitutions abolished antifungal activity. In contrast, most residues in loop 1B could be modified without abolishing activity and substitution of residue 28 in loop 4 decreased but did not abolish activity (**Figure 3**). NaD1 was chosen for modification over NaD1-L1B-HRFKGP as L1B from NaD1 has an extra predicted pepsin cleavage site.

On the basis of this analysis, we decided to substitute loop 1B with the pepsin cleavage site from lactoferrin RAFALE and to make loop 4 a better pepsin site with a lysine 28 to phenylalanine substitution.

**Figure 4:**
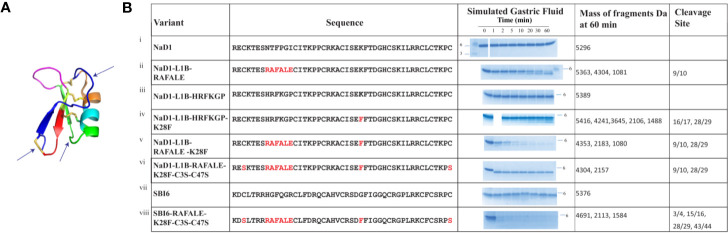
Digestion of NaD1, NaD1-L1B-HRFKGP, SBI6, and their variants in the SGF assay. **(A)** Arrows indicate locations in the NaD1 structure where modifications were made. The structure was adapted from PDB 1MR4. **(B)** The names and sequences of NaD1 and the variants listed next to gel images showing products obtained in the SGF assay. Incubation time is indicated above the lanes and the position of the 6 kDa size marker is marked. Unmodified NaD1 (i) NaD1-L1B-HRFKGP (iii) and SBI6 (vii) were not degraded in SGF. All variants were either partially or completely digested. The size of the pepsin cleavage products is presented as well as the positions where the cleavage occurred.

The effects of these changes on pepsin digestibility are illustrated in **Figure 4**. The NaD1-L1B-RAFALE variant was slightly more susceptible to pepsin with some breakdown at 60 min (**Figure 4**). Cleavage occurred between amino acid position 9/10 producing fragments of 4304 Da and 1081 Da. However, full-length protein was still present at 60 min. The exchange of lysine with a phenylalanine at position 28 also improved digestion by pepsin (**Figure 4**). Fragments of 4241, 3645, 2106, 1488 appeared at 10 min coinciding with cleavage between residues 16/17 and 28/29. However, full-length protein remained for up to 60 min. The variant with the combined loop 1B and loop 4 substitutions was completely converted into fragments of 4304 Da and 2157 Da within 30 min (**Figure 4**) consistent with pepsin cleavage between residues 9/10 and 28/29. Further substitution of cysteine 3 and cysteine 47 from loop 1A and loop 7 respectively with serine residues, to remove the disulfide bond that forms the stabilizing pseudo-cyclic structure, was performed on the loop 1B/loop 4 substitution variant. This variant was completely broken down within 1 minute into fragments of 4304 Da and 2157 Da (**Figure 4**) consistent with cleavage between residues 9/10 and 28/29. A similar improvement in pepsin digestibility was obtained (**Figure 4**) when the same modifications were introduced into SBI6 which shares 36% sequence identity with NaD1.

**Figure 5:**
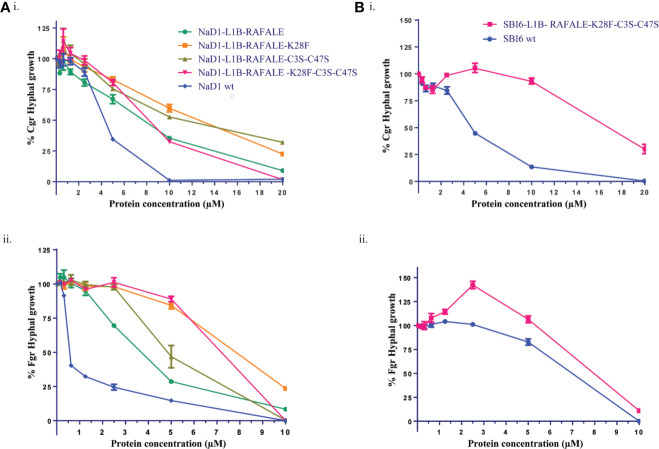
Digestible variants of NaD1 and SBI6 inhibit the growth of *C. graminicola* and *F. graminearum*. **(A)** Antifungal activity of digestible NaD1 variants against (i) *F. graminearum* and (ii) *C. graminicola* plotted as growth relative to an untreated control. **(B)** Antifungal activity of digestible SBI6 variants against (i) *F. graminearum* and (ii) *C. graminicola* plotted as growth relative to an untreated control. Variants of both defensins retained activity against both pathogens although it was diminished. Graphs are representative of data from three separate experiments performed with two technical replicates. Error bars represent standard error of the mean.

The effect of these modifications on antifungal activity was tested using growth assays with *F. graminearum* and *C. graminicola*. The SBI6 and NaD1 variants were less active than the wild type defensins, but still inhibited the growth of both fungi at 20 µM (**Figure 5**).

**Figure 6:**
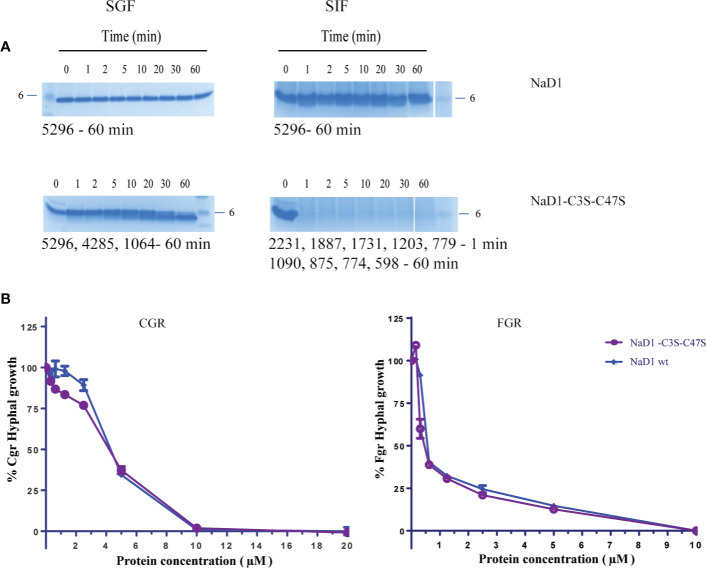
Comparison of the NaD1-C3S-C47S variant lacking the fourth disulfide bond and native NaD1 in the SGF and SIF digestion assays and antifungal activity assays. Time course of digestion of **(A)** native NaD1 and NaD1-C3S-C47S variant in the SGF and SIF assays. Incubation time is indicated above the lanes and the 6 kDa size marker is marked. The variant was partially digested in the SGF and completely digested within 1 min in SIF. The size of fragments is displayed under each gel. **(B)** Growth of *C*. *graminicola* and *F. graminearum* in the presence of the NaD1-C3S-C47S variant or native NaD1 plotted relative to a no-protein control. Graphs are from a single experiment with two technical replicates but are representative of data from three separate experiments. Error bars represent standard error of the mean.

We then asked whether removal of the Cys3-Cys47 disulfide bond alone would be sufficient to enhance digestibility. NaD1-C3S-C47S was partially digested in the SGF assay by 30 min. However, when digestion products are larger than 3.5 kDa, they may pass the digestibility requirements if they are rapidly broken down in the simulated intestinal fluid assay (SIF). NaD1 and the NaD1-C3S-C47S variant were thus subjected to trypsin in the SIF assay. NaD1 was not digested by SIF but NaD1- C3S-C47S was completely digested by SIF within 1 minute (**Figures 6A, B**). The NaD1-C3S-C47S variant was as active as unmodified NaD1 in growth assays with *F. graminearum* and *C. graminicola* (**Figure 6C**).

#### Accumulation of Digestible NaD1 Variants *in Planta*

**Figure 7:**
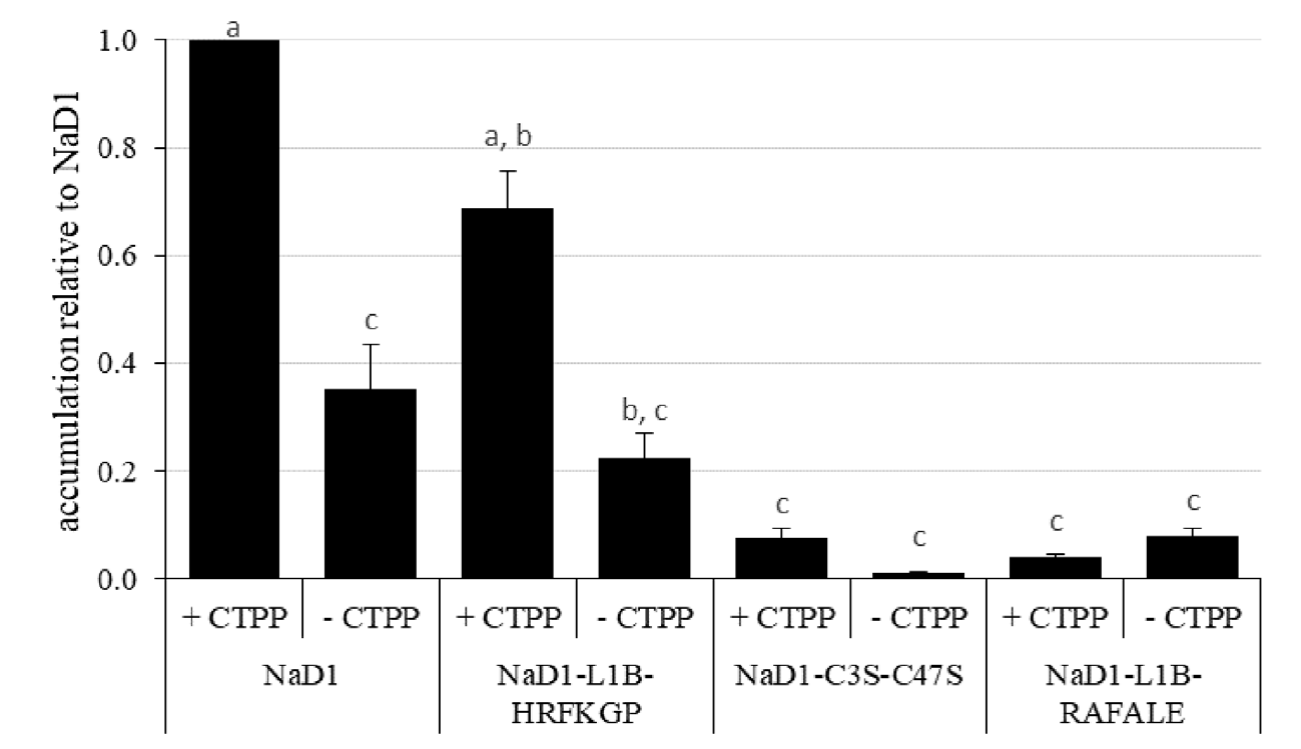
Relative accumulation of NaD1 and variants with and without the NaD1 C-terminal propeptide (CTPP) in bush bean cotyledons after Agroinfiltration. Accumulation relative to NaD1 + CTPP for each of the other variants (± CTPP) was calculated for each plant (n = 4) and then averaged (± SEM). Accumulation data for each plant is shown in . Different letters indicate significant differences found by Tukey’s ANOVA (P<0.05) (all P values are shown in ).

We then investigated whether the more digestible NaD1 variants could accumulate to similar levels *in planta* as the parent defensin. This analysis was conducted using transient expression in bush bean cotyledons (**Figure 7**). After 5 days post-infiltration, NaD1 accumulation ranged from 29 to 64 ppm when produced with its cognate C-terminal propeptide (**Supplementary Figure 1**). NaD1-L1B-HRFKGP, which like NaD1 is stable in the SGF assay, accumulated at levels about 69% of NaD1. In contrast, the NaD1 variants with improved digestibility accumulated poorly, with NaD1-C3S-C47S and NaD1-L1B-RAFALE only reaching between 7% and 4% of NaD1 levels, respectively. There was a similar trend when proteins were produced without the C-terminal propeptide. When the empty vector control was transiently expressed, the ELISA signal was below the limit of quantitation.

## Discussion

Several research groups have used plant defensins to produce transgenic crop plants that are more resistant to fungal diseases (Gaspar et al., 2014; Bala et al., 2016; Sasaki et al., 2016; Kaur et al., 2017). However, before any of these plants can be released commercially they must pass a series of regulatory requirements defined in the FAO/WHO decision tree (Metcalfe et al., 1996; FAO/WHO, 2001) to ensure that the defensins are not potential allergens. In this study, we examined two well-known defensins to determine whether they would pass the allergenicity and digestibility tests outlined in the decision tree. That is, did they share sequence with any known allergens and were they rapidly degraded into fragments of less than 3.5 kDa in simulated gastric- or intestinal fluid assays? The two defensins, NaD1 from the solanaceous plant, *Nicotiana alata* and SBI6 from soybean were not related to any known allergens apart from three defensins from peanuts (Petersen et al., 2015) which have up to 26% and 40% sequence identity respectively but no more than two contiguous identical residues (**Figure 1**). All plant defensins share at minimum 19%–26% sequence identity to the peanut defensins, AraH12, 13.1, and 13.2 due to conservation of the eight cysteine and two glycine residues that are essential for the defensin fold. Thus, most of the sequence identity identified between our two defensins and peanut defensins is due to these conserved residues. While peanut defensins are not the dominant peanut allergens, a subset of patients with severe allergy to peanuts have IgE in their sera that cross reacts with peanut defensins (Petersen et al., 2015). Perhaps any plant defensins that were going to be used for a commercial transgenic plant could be tested for reactivity against sera from people with peanut allergies.

The databases of potential allergens are continually updated. For example, the database Allergenonline, (http://Allergenonline.org v19) held only 219 allergens in 1996 (Goodman et al., 2005) and had increased to 2129 in 2019. Proteins are deemed potential allergens, if they share eight contiguous amino acids with a known allergen. NaD1 and SBI6 did not have any matching stretches of eight amino acids. However, a single short identity match of only six to eight contiguous amino acids is not the best indicator of potential allergenicity, as the test protein and the allergen are unlikely to share IgE epitopes. Sequence identity to an allergen of 35% or greater over a length of 85 residues is a more reliable predictor, but this does not apply to defensins which are only 45–54 residues in length (Herman et al., 2009; Goodman et al., 2016).

For effective antibody binding, an allergen must have at least two IgE binding sites that are each at least 15 amino acids long (Moreno, 2007). NaD1 and SBI6 defensin sequences were tested for predictions in IgE binding sites including the peanut defensins. Neither was identified as having experimentally proven IgE binding sites. This was anticipated as most plants, particularly seeds, have defensins and many plant defensins are consumed daily without complication. Conformational epitopes (antibody epitopes that involve amino acids that are separated in the primary protein sequence but sit close together in the three-dimensional structure) are generally associated with airborne allergens like pollen and spores (aeroallergen-mediated allergic reactions) and not food allergens (Bannon, 2004). Three proteins with defensin-like domains have been described as pollen antigens, that is, aeroallergens. They are Art v1 from mugwort, Amb a 4 from ragweed and Par h 1 from feverfew (Himly et al., 2003; Léonard et al., 2010; Pablos et al., 2018). The sequence identity of NaD1 and SBI6 with the defensin domains of the aeroallergens is between 19%–28% (NaD1 27, 26, 28, and SBI6 19, 21, 26% respectively).

Both NaD1 and SBI6 were very stable in the simulated gastric and intestinal fluid assays (**Figure 6**). Stability to pepsin in the SGF assay was initially established as a good predictor for food allergens because many important food allergens are stable upon exposure to pepsin at low pH (1.2) (Astwood et al., 1996) and stable proteins or protein fragments are more likely to be presented to the immune system. This is the basis for the WHO requirement for products of transgenes to be rapidly degraded in a simulated gastric fluid and/or simulated intestinal fluid assay.

Despite having four predicted pepsin cleavage sites and seven trypsin sites, NaD1 was completely stable in the presence of pepsin and trypsin (**Figure 6**). SBI6, with five predicted pepsin cleavage sites, was also not digested by pepsin (**Figure 2**). These observations led us to introduce a series of modifications to increase the digestibility of plant defensins to enable them to comply with WHO requirements.

The fact that none of the four predicted pepsin cleavage sites in NaD1 are hydrolyzed by pepsin indicates that the compact tertiary structure of defensins prevents accessibility of the protease to the cleavage site or that the rigidity of the protein orients the amino acid side chains in a way that is not amenable to binding to the pepsin active site. Thus, we introduced pepsin cleavage sites to more exposed and/or flexible regions of the molecule. These variants also needed to retain their antifungal activity to be useful. Three exposed, flexible loops (loop 1B, loop 4, and loop 5) were considered for mutagenesis. We knew that loop 1B was amenable to changes from a previous analysis of *N. alata* defensin chimeras that identified an improved defensin NaD1-L1B-HRFKGP (Bleackley et al., 2016). A library of single amino acid substitutions in the three loops was screened to assess their effect on activity against the plant pathogens *F. graminearum* and *C. graminicola*. NaD1-L1B-HRFKGP is identical to NaD1 apart from five residues in loop 1B. Unexpectedly, this substitution led to a 2-fold increase in activity against *F. graminearum* and *C. graminicola* and ten-fold enhanced activity against the fungal pathogen *F. oxysporum* (Bleackley et al., 2016). Thus, we surmised that loop 1B of NaD1 could be modified to incorporate a pepsin cleavage site while retaining antifungal activity. A single amino acid substitution library of NaD1-L1B-HRFKGP was designed to identify additional residues that are amenable to change without affecting the antifungal activity as well as residues that improve antifungal activity (**Figure 3**). As expected, loop 1B tolerated changes well, while changes in loop 4 only caused a minor reduction in activity. In contrast, single amino acid substitutions in loop 5 abolished activity against *F. graminearum* and *C. graminicola* supporting its essential role in antifungal activity (Van Der Weerden et al., 2013; Meindre et al., 2014; Poon et al., 2014).

Based on these data, two new pepsin cleavage sites were introduced into loop 1B (RAFALE) and loop 4 (K28F). The RAFALE sequence was chosen from lactoferrin because it is a natural pepsin substrate. This sequence sits adjacent to a cysteine residue in lactoferrin, so it was likely to be tolerated next to a cysteine residue and a disulfide bond in the defensin. The K28F substitution next to F29 introduced an FF site which is a preferred pepsin cleavage site. Cleavage at this site would assist in breaking down the molecule to fragments of less than 3.5 kDa. Pepsin did cleave at both of the introduced pepsin sites, RAFALE and K28F in NaD1 and NaD1-L1B-HRFKGP respectively even though the predicted natural pepsin sites in native NaD1 at position 10 (Phe) and 11 (Pro) and 28 (Lys) and 29 (Phe) were not cleaved (**Figure 4**). However, cleavage of these two variants was still too slow for regulatory purposes because some full-length protein remained at 30 and 60 min respectively. Combining the two modifications into the one variant further reduced digestion time but did not meet regulatory requirements.

This led to the decision to remove the disulfide bond that connects the N- and C- termini of plant defensins because this bond increases stability by making them pseudo-cyclic. In addition, this disulfide bond is on the opposite side of the molecule to loop 5 which is essential for antifungal activity and it is not likely to be essential for folding because it is absent in the mammalian β-defensins and the insect defensins (Shafee et al., 2016). Replacing the cysteine residues at positions 3 and 47 with serine residues in combination with the two introduced pepsin cleavage sites (RAFALE and K28F) produced a defensin variant that was hydrolyzed within 1 min in the gastric fluid assay and produced digestion products below the regulatory stipulated size 3.5 kDa (**Figure 4**). This was a vast improvement to the variant with just the two new pepsin sites (RAFALE and K28F). No smaller fragments were produced over time indicating the predicted pepsin cleavage sites in the native molecule were still not suitable substrates.

Removal of the disulfide bond from NaD1 without the RAFALE and K28F modifications was not sufficient to meet the time requirement for complete digestion in the SGF assay, but it was sufficient for complete digestion in the SIF assay. There are seven trypsin cleavage sites in NaD1 (positions 1, 4, 22, 28, 29, 36, 39, 40) which were not susceptible to cleavage when the four disulfide bonds are intact. The disulfide variant was digested into peptides of less than 2300 Da in SIF within 1 min consistent with cleavage at all seven trypsin cleavage sites. Thus, removal of just the fourth disulfide bond may be sufficient to meet the regulatory requirements.

Incorporation of the RAFALE, K28F, and C3S-C47S variations into the SBI6 backbone also converted the defensin from a molecule that was stable in SGF for more than 60 min into a variant that was completely digested within 5 min according to the SDS-PAGE assay (**Figure 4**). A small amount of full-length protein was still detectable after 10 min when mass spectrometry was used for analysis. It is likely that this small amount of full-length protein would be rapidly broken down in SIF.

The antifungal activity of all the digestible variants for both NaD1 and SBI6, apart from the variant with only the terminal disulfide removed, was lower than the parent defensins against *F. graminearum* and *C. graminicola*. Altogether, nine residues (19%) were modified to create the NaD1/SBI6-L1B-RAFALE-K28F-C3S-C47S variants to achieve the goal of complete digestion in simulated gastric fluid within 1 min. However, improved digestibility came at the expense of potent antifungal activity (IC_50_ 0.5 µM for NaD1) with up to a 14-fold reduction against *F. graminearum* and about a 4-fold reduction against *C. graminicola* for the NaD1-L1B-RAFALE-K28F-C3S-C47S variant (**Figures 5A**). Interestingly, the opposite was observed for the SBI6-RAFALE-K28F-C3S-C47S variant as up to a 13-fold reduction was observed against *F. graminearum* and only a single fold reduction in activity against *C. graminicola* (**Figures 5B**). Interestingly, when only the fourth disulfide bond was removed, the antifungal activity of the molecule was unchanged (**Figure 6**). This suggests that this highly conserved feature of plant defensins has evolved to enhance the stability of the molecule and is less important for antifungal activity.

As well as retaining antifungal activity, defensins that have been engineered for increased digestibility must also accumulate *in planta* if they are to confer disease resistance in transgenic crops. However, the two digestible NaD1 variants tested in this study accumulated at much lower levels than the parent defensin in a bush bean transient expression system. This occurred whether they were produced with their cognate C-terminal propeptide, which targets them to the vacuole, or without, which results in accumulation in the apoplast (Lay et al., 2014). If the relative accumulation levels of the digestible variants were replicated in stable transformants, it is doubtful that antifungal activity would be conferred. We hypothesize that enhancing the *in vitro* digestibility of NaD1 also increases its susceptibility to proteases *in planta*. Further experiments to mitigate this effect could include targeting the defensin to a different cell compartment or tissue type or using a stronger promoter to express the defensin. How widespread this effect is amongst different plant species also remains to be seen but we have obtained similar results for the accumulation of NaD1-L1B-RAFALE in stably transformed corn plants where the average accumulation in leaf sheath was only around 3% of the average accumulation of NaD1 (data not shown).

In conclusion, we have identified residues that can be engineered into a defensin backbone to promote digestibility in the simulated gastric fluid assay. The most promising variant was the NaD1- C3S-C47S mutant. Removal of the disulfide bond that joins the N- and C-termini alone improved digestibility with both pepsin and pancreatin and did not alter the antifungal activity. This simple change is likely to enhance the digestibility of other plant defensins and may help them reach their commercial potential. However, enhanced digestibility may also affect their accumulation *in planta* and therefore the effectiveness of any conferred trait. This is another hurdle that must be overcome before plant defensins can be used commercially in transgenic plants.

## Data Availability Statement

The original contributions presented in the study are included in the article/**Supplementary Material**; further inquiries can be directed to the corresponding author.

## Author Contributions

KP, MB, MA, and NW conceived and planned the experiments and contributed to the interpretation of the results. KP carried out the *in silico* analyses, simulated digestion assays and antifungal experiments. SP planned and carried out the accumulation of defensins *in planta* experiments. RR and GS contributed to sample preparation. KP and MA took the lead in writing the manuscript. All authors contributed to the article and approved the submitted version.

## Funding

This project was conducted with funds provided by Hexima Ltd.

## Conflict of Interest

The authors declare that this study receivedfundingfrom Hexima Ltd.. The funder was not involved in the study design, collection, analysis, interpretation of data, the writing of this article or the decision to submit it for publication. Authors JE and NY were employed by company Corteva Agriscience.

The remaining authors declare that the research was conducted in the absence of any commercial or financial relationships that could be construed as a potential conflict of interest.

## Supplementary Material

Supplementary Figure 1 Accumulation of NaD1 and variants with and without the NaD1 C-terminal propeptide (CTPP) in bush bean cotyledons after Agroinfiltration. Accumulation of NaD1 and variants in plants A. with the vacuole targeting C-terminal propeptide and B. without the C-terminal propeptide.

Supplementary Figure 2 Statistical comparison of mean transient expression for all constructs. Boxes highlighted in yellow indicate constructs with statistically significant differences in mean expression. P <0.05, Tukey’s HSD.
