# Supplementary figures and images for: Improving the Digestibility of Plant Defensins to Meet Regulatory Requirements for Transgene Products in Crop Protection
Source: Front Plant Sci. 2020 Aug 14;11:1227. doi: 10.3389/fpls.2020.01227 (PMC7456892; doi:10.3389/fpls.2020.01227)

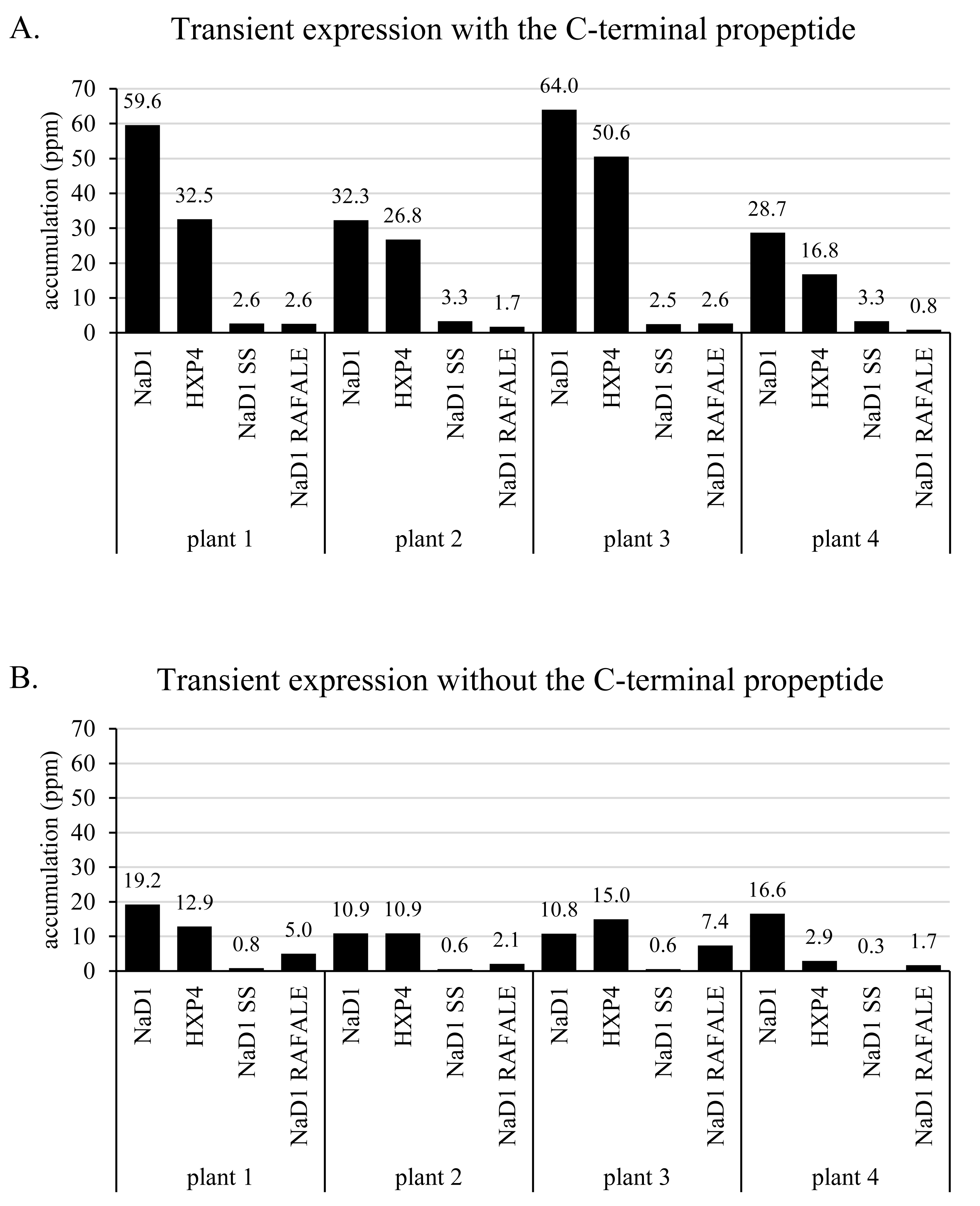

Supplement: Supplementary Figure 1 — Accumulation of NaD1 and variants with and without the NaD1 C-terminal propeptide (CTPP) in bush bean cotyledons after Agroinfiltration. Accumulation of NaD1 and variants in plants A. with the vacuole targeting C-terminal propeptide and B. without the C-terminal propeptide. [file Image_1.tif]

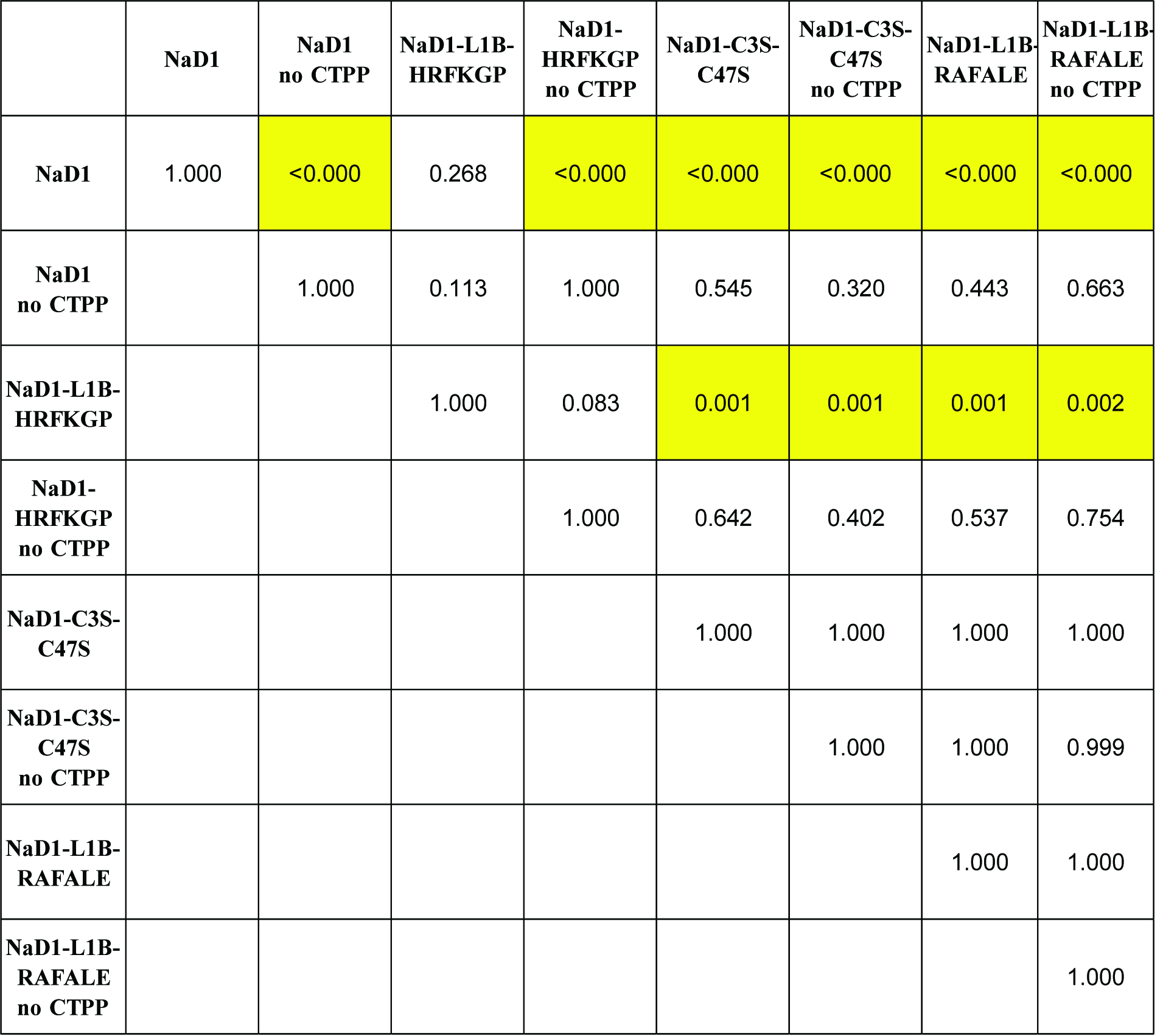

Supplement: Supplementary Figure 2 — Statistical comparison of mean transient expression for all constructs. Boxes highlighted in yellow indicate constructs with statistically significant differences in mean expression. P <0.05, Tukey’s HSD. [file Image_2.tif]
